# Vitamin D Deficiency and Mortality in Pediatric Dialysis: Potential Confounding and Reverse Causality

**DOI:** 10.1002/hsr2.72697

**Published:** 2026-06-19

**Authors:** Sami Ullah Khan, Syed Huzaifa Khan, Muhammad Faizan Khan

**Affiliations:** ^1^ Nowshera Medical College Nowshera Khyber Pakhtunkhwa Pakistan; ^2^ Khyber Medical University Peshawar Kyber Pakhtunkhwa Pakistan

Dear Editor,

We read with great interest the recent article by Khamene et al. [1] examining the association between serum vitamin D levels and mortality in children undergoing chronic dialysis. The authors are to be commended for addressing an important and vulnerable population, particularly given the high prevalence of vitamin D deficiency in pediatric end‐stage renal disease (ESRD). Their finding of a hazard ratio of 3.2 for mortality in children with vitamin D levels < 15 ng/mL is clinically striking and warrants thoughtful discussion.

However, we would like to highlight two methodological considerations that may temper causal interpretation.

First, residual confounding by underlying illness severity may partially explain the observed association. Children with more advanced end‐stage renal disease (ESRD), systemic inflammation, malnutrition, or greater comorbidity burden are biologically predisposed to both lower 25‐hydroxyvitamin D [25(OH)D] levels and higher mortality risk. Observational studies in broader populations have consistently demonstrated associations between low vitamin D and mortality, while acknowledging the possibility of residual confounding despite multivariable adjustment. For example, Melamed et al. reported an independent association between low 25(OH)D and mortality in the general population but emphasized limitations inherent to observational design [2]. More importantly, a large systematic review and meta‐analysis by Chowdhury et al. found that although observational studies suggested strong mortality associations, randomized controlled trials demonstrated substantially weaker and less consistent survival benefits [3]. This discrepancy raises concern that low vitamin D may function primarily as a marker of poor overall health status rather than a direct causal determinant of mortality.

Second, reverse causality is particularly plausible in dialysis populations. Chronic kidney disease alters vitamin D metabolism through impaired hydroxylation, inflammation, protein‐energy wasting, reduced mobility, and decreased sunlight exposure. Consequently, lower vitamin D levels may represent a downstream effect of advanced disease rather than its cause. Autier et al., in a systematic review, argued that vitamin D deficiency frequently reflects underlying ill health, and that reverse causation likely explains many associations reported in observational studies [4]. In pediatric ESRD, where metabolic disturbances and inflammatory burden are intrinsic to disease progression, this possibility deserves careful consideration.

Additionally, the retrospective design and relatively small sample size (*n* = 53) further limit causal inference. Although multivariate Cox regression was performed, residual confounding cannot be fully excluded, particularly in complex conditions such as ESRD where nutritional status, inflammatory markers, dialysis adequacy, and comorbidity burden are interrelated.

We fully agree that monitoring vitamin D levels is appropriate in pediatric dialysis care and aligns with established CKD–mineral bone disorder management strategies. However, statements suggesting that vitamin D deficiency “significantly impacts survival” or that routine supplementation may directly improve mortality risk should be interpreted cautiously in the absence of randomized interventional evidence demonstrating survival benefit in this population.

Future prospective studies with larger cohorts, repeated vitamin D measurements, adjustment for detailed nutritional and inflammatory indices, and ideally randomized supplementation trials would help clarify whether vitamin D is a modifiable causal factor or primarily a prognostic biomarker.

We appreciate the authors' contribution to this important field and hope that this discussion supports continued rigorous investigation into improving outcomes for children with ESRD.

## Author Contributions

**Sami Ullah Khan:** investigation, conceptualization, writing – original draft. **Syed Huzaifa Khan:** validation, writing – review and editing, formal analysis, supervision. **Muhammad Faizan Khan:** writing – review and editing, validation, formal analysis, supervision.

## Funding

The authors have nothing to report.

## Disclosure

The corresponding author (Sami Ullah Khan) affirms that this manuscript is an honest, accurate and transparent account of the study being reported; that no important aspects of the study have been omitted; and that any discrepancies from the study as planned have been explained.

## Ethics Statement

The authors have nothing to report.

## Consent

The authors have nothing to report.

## Conflicts of Interest

The authors declare no conflicts of interest.

## Data Availability

Data sharing not applicable to this article as no datasets were generated or analyzed during the current study. No new data was generated in this work.
